# Author response to Comment on: Cytokines (IL1β, IL6, TNFα) and serum cortisol levels may not constitute reliable biomarkers to identify individuals with post-acute sequelae of COVID-19

**DOI:** 10.1177/17562864241255819

**Published:** 2024-05-31

**Authors:** Michael Fleischer, Fabian Szepanowski, Anne K. Mausberg, Livia Asan, Ellen Uslar, Denise Zwanziger, Mark Stettner, Lothar Volbracht, Christoph Kleinschnitz

**Affiliations:** Department of Neurology and Center for Translational and Behavioral Neurosciences, University Medicine Essen, University of Duisburg–Essen, Essen, Germany; Department of Neurology and Center for Translational and Behavioral Neurosciences, University Medicine Essen, University of Duisburg–Essen, Essen, Germany; Department of Neurology and Center for Translational and Behavioral Neurosciences, University Medicine Essen, University of Duisburg–Essen, Essen, Germany; Department of Neurology and Center for Translational and Behavioral Neurosciences, University Medicine Essen, University of Duisburg–Essen, Essen, Germany; Department of Neurology and Center for Translational and Behavioral Neurosciences, University Medicine Essen, University of Duisburg–Essen, Essen, Germany; Clinical Chemistry – Division of Laboratory Research, Department of Endocrinology, Diabetes and Metabolism, University Medicine Essen, University of Duisburg–Essen, Essen, Germany; Department of Neurology and Center for Translational and Behavioral Neurosciences, University Medicine Essen, University of Duisburg–Essen, Essen, Germany; Central Laboratory, University Medicine Essen, University of Duisburg–Essen, Essen, Germany; Department of Neurology and Center for Translational and Behavioral Neurosciences, University Medicine Essen, University of Duisburg–Essen, Hufelandstraße 55, Essen 45147, Germany

Dear Editor,

We would like to express our gratitude to the author for his interest in our scientific work^1^ and his thorough review. In the following, we address the points of criticism mentioned.

Indeed, the current study was designed to be prospective and observational, as was our previously published study on the (missing) consequences of post-COVID-19: coronavirus disease (COVID) on the nervous system.^2^ Also, both studies were assigned the same local ethics committee registration number (20-9284-BO, 20-9307-BO). It is common practice in Germany to conduct different sub-studies on the same topic, that is, clinical outcomes *versus* biomarker studies, under one ethics application.

The author further criticizes that the study was not deposited in a registry for clinical studies and claimed that Strengthening the reporting of observational studies in epidemiology (STROBE) guidelines have not been considered. However, preregistration of such studies is neither mandatory nor does the publishing journal demand such preregistrations. In fact, the vast majority of observational studies are not preregistered. The STROBE guidelines were created to aid authors in presenting their work and not to act as a validation tool or as a framework for the design of observational studies. The core elements proposed in the STROBE guidelines, such as a transparent and easily accessible presentation of the objectives and findings, are obviously present in our work; likewise, we have clearly stated the limitations of our study findings in the manuscript.

Regarding the author’s criticism on the lack of sample size calculations, we want to emphasize that all available patients who met the enrolment criteria were recruited into the study as stated in the Materials and Methods section. The ratio between individuals with ongoing post-acute sequelae of severe acute respiratory syndrome coronavirus 2 (SARS-CoV-2) infection (PASC) and controls that never had a SARS-CoV-2 infection or never developed PASC was similar to that reported by Klein *et al*.^3^ Of note, we in the meantime have analysed cortisol and cytokines levels in a considerable number of additional patients suffering from post-COVID in our large outpatient centre and were unable to find any abnormalities.

Furthermore, the author calls into question the prospective nature of our study as well as the original study objectives. Indeed, the study started to recruit patients in early 2021. As some of the symptoms reported by the patients suggested the relevance of soluble immune mediators or an endocrinological deficit (e.g. fatigue, cognitive deficits) as the underlying cause, we determined these parameters already at an early stage and collected blood samples as part of the study. Later on, only data sets from post-COVID patients who met the World Health Organization (WHO) Delphi consensus criteria were analysed and included into the present study.

Regarding the statistical analysis, we agree that with *F* tests in multiple comparisons, such as Kruskal–Wallis–ANOVA, the *post hoc* test is not required when the initial test does not show any significant difference between multiple groups. We follow a routine procedure when analyzing data to test for normal distribution in a data set in our lab, even if reporting is not strictly required. Of note, we also analyzed the data using the more robust multiple comparison tests for unequal variances, such as Welch’s ANOVA, which led to similar results. Moreover, we also applied Kruskal–Wallis–ANOVA after removing outliers; however, no significant difference was found. Hence, regardless of the statistical approach applied, the key results remained unchanged, and we could not find any relevant differences in cytokine or cortisol levels between the groups. In summary, we applied a rigorous study design including utilization of the same testing kits as in the reference studies^3,4^ in four well-defined patient groups to minimize bias and to exclude that our conclusions are based on false-negative results.

Taken together, our study demonstrated that neither serum levels of Interleukin-1 beta (IL-1β), Interleukin-6 (IL-6), and Tumor necrosis factor alpha (TNFα), nor cortisol are suitable biomarkers to identify patients with PASC. Interestingly, these ‘negative’ findings have recently been confirmed by other groups^5–7^ underlining the urgent need of independent replication of results in post-COVID research. Given the lack of valid biomarkers and relevant organ damage,^2,5,8,9^ the conclusion that PASC might represent a primarily psychosomatic or functional disorder is, therefore, reasonable and appropriate.

## Declarations

**Ethics approval and consent to participate:** Not applicable.

**Consent for publication:** Not applicable.

**Availability of data and materials:** Not applicable.
